# SAM68: Signal Transduction and RNA Metabolism in Human Cancer

**DOI:** 10.1155/2015/528954

**Published:** 2015-07-26

**Authors:** Paola Frisone, Davide Pradella, Anna Di Matteo, Elisa Belloni, Claudia Ghigna, Maria Paola Paronetto

**Affiliations:** ^1^Laboratory of Cellular and Molecular Neurobiology, Santa Lucia Foundation, 00143 Rome, Italy; ^2^Institute of Molecular Genetics-National Research Council (IGM-CNR), 27100 Pavia, Italy; ^3^University of Rome “Foro Italico”, Piazza Lauro de Bosis 15, 00135 Rome, Italy

## Abstract

Alterations in expression and/or activity of splicing factors as well as mutations in *cis*-acting splicing regulatory sequences contribute to cancer phenotypes. Genome-wide studies have revealed more than 15,000 tumor-associated splice variants derived from genes involved in almost every aspect of cancer cell biology, including proliferation, differentiation, cell cycle control, metabolism, apoptosis, motility, invasion, and angiogenesis. In the past decades, several RNA binding proteins (RBPs) have been implicated in tumorigenesis. SAM68 (SRC associated in mitosis of 68 kDa) belongs to the STAR (signal transduction and activation of RNA metabolism) family of RBPs. SAM68 is involved in several steps of mRNA metabolism, from transcription to alternative splicing and then to nuclear export. Moreover, SAM68 participates in signaling pathways associated with cell response to stimuli, cell cycle transitions, and viral infections. Recent evidence has linked this RBP to the onset and progression of different tumors, highlighting misregulation of SAM68-regulated splicing events as a key step in neoplastic transformation and tumor progression. Here we review recent studies on the role of SAM68 in splicing regulation and we discuss its contribution to aberrant pre-mRNA processing in cancer.

## 1. Introduction

SAM68 (SRC associated in mitosis of 68 kDa) was originally identified as a protein physically associated with and phosphorylated by the tyrosine kinase c-SRC during mitosis [1, 2], opening the interesting possibility of a signaling circuitry driven by c-SRC and affecting RNA processing and trafficking in a cell-cycle-dependent manner.

**Figure 1:**
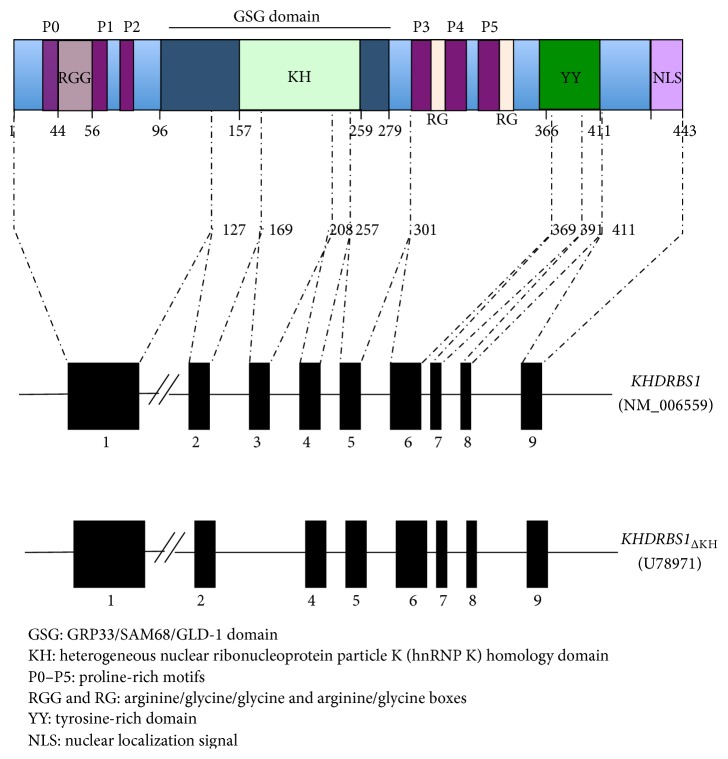
Schematic representation of SAM68 domains. In the upper part, schematic model representing the structural/functional domains of SAM68 protein as a prototype of a STAR protein. SAM68 protein is composed of the GRP33/SAM68/GLD-1 (GSG) domain, formed by a single heterogeneous nuclear ribonucleoprotein particle K (hnRNP K) homology domain (KH) embedded in two flanking regions, six consensus proline-rich motifs (P0–P5), arginine/glycine/glycine (RGG) and arginine/glycine (RG) boxes, C-terminal tyrosine-rich domain (YY), and a nuclear localization signal (NLS). In the lower part, the two protein coding mRNA isoforms of human* KHDRBS1* are represented. Black boxes indicate exons (numbered from 1 to 9). The sizes of exons and the protein domains encoded by each exon are indicated.

SAM68 belongs to the STAR (signal transduction and activation of RNA metabolism) family of RNA binding proteins (RBPs) that link signaling pathways to RNA processing [3, 4]. STAR proteins include* Artemia salina* GRP33 [5],* C. elegans* GLD-1 [6], mammalian QKI [7], SAM68 [8, 9], SLM-1 and SLM-2 [10, 11],* Drosophila* HOW [12], KEP1 and Sam50 [13], and the evolutionary conserved splicing factor SF1 [14]. All STAR proteins, from worms to mammals, share common architecture (Figure 1). They contain a GRP33/SAM68/GLD-1 (GSG) domain for RNA binding and homodimerization, flanked by regulatory regions harboring motifs for protein-protein interactions (Figure 1), often mediated by conserved amino acid residues targeted by posttranslational modifications [15]. SAM68 contains six proline-rich sequences and a tyrosine-rich region at the C-terminus, which form docking sites for signaling proteins containing SRC homology 3 (SH3) and 2 (SH2) domains (Figure 1) [1, 2, 9, 16]. Notably, tyrosine phosphorylation by SRC-related kinases impairs SAM68 homodimerization [17] as well as its affinity for RNA both* in vitro* [16, 18] and* in vivo* [19]. Additional posttranslational modifications were also reported to affect the functions of this RBP. SAM68 binds to and is methylated by the arginine methyltransferase PRMT1 [20], thus affecting SAM68 interaction with SH3 domains [21] and its nuclear localization [20]. SAM68 acetylation, described in tumorigenic breast cancer cell lines [22], by the acetyltransferase CBP increases SAM68 binding to RNA* in vitro*. Furthermore, SAM68 can be SUMOylated by the SUMO E3 ligase PIAS1, which enhances its transcriptional repression activity [23]. Thus, posttranslational modifications greatly influence the biochemical properties of SAM68 and finely tune its subcellular localization, interaction with signaling proteins, and RNA binding affinity.

Despite the growing interest in STAR proteins, their physiological role has not been completely elucidated yet. Nevertheless, recent mouse models of genetic ablation of STAR proteins are now greatly helping in pursuing this goal. In this review, we discuss the functional properties of SAM68 in signaling and RNA metabolism, with particular emphasis on malignant transformation. In particular, we highlight recent advances and new insights into SAM68-based signaling that have been made in the last two decades, which expand our understanding of STAR-mediated signaling in cancer cells.

## 2. SAM68 Biological Role(s): Lessons from Mouse Models

The first indication of the involvement of STAR proteins in tumorigenesis came from studies in* C. elegans*. Critical missense mutations in the* gld-1* gene caused germ-line tumors, thus suggesting an important role for* GLD-1* as a tumor suppressor [24]. These null mutations in hermaphrodites caused female germ cells to exit from the meiotic prophase and to start proliferating, thus leading to the formation of a germ-line tumor [3]. In this regard, it is important to notice that the function and localization of GLD-1 appear quite different from the SAM68 subfamily of STAR proteins. Indeed, GLD-1 is localized exclusively in the cytoplasm of germ cells and it does not contain the protein domains flanking the GSG of SAM68, which are involved in cell signaling [3]. Nevertheless, an initial observation seemed to suggest a similar tumor suppressor role also for SAM68. A random homozygous knockout (RHKO) screen in NIH3T3 murine fibroblasts indicated that functional inactivation of the* Sam68* gene induces tumorigenesis and allows NIH3T3 cells to form metastatic tumors in nude mice [25]. These studies suggested that SAM68 negatively affects neoplastic transformation, like its* C. elegans* ortholog* GLD-1*. However, in contrast to this proposed function, disruption of the* Sam68* gene in chicken DT40 cells showed reduced growth rate, indicating that SAM68 plays a positive role in cell proliferation [26]. Moreover, a natural alternative isoform of* SAM68* with deletion of the KH (RNA binding) domain (*SAM68*_ΔKH_) was specifically expressed during growth arrest in normal cells, but absent in SRC-transformed cells (Figure 1) [27]. Importantly, transfection of the* SAM68*_ΔKH_ isoform inhibited serum-induced DNA synthesis and Cyclin D1 expression, thus highlighting for the first time the involvement of SAM68 RNA binding activity in cell proliferation [27]. Thus, despite the initial putative role as a tumor suppressor gene, subsequent studies appeared to suggest a positive role of SAM68 in tumorigenesis. These findings were also supported by investigation of the* Sam68* knockout mouse model, which has recently unveiled the physiological processes in which SAM68 is involved.

*Sam68*-deficient mice displayed high lethality soon after birth [28]. Nevertheless, mice that survived beyond weaning showed a normal lifespan. Importantly, surviving* Sam68−/−* mice lived to old age (~2 years) and were not prone to tumor formation, clearly indicating that SAM68 is not a tumor suppressor* in vivo* [28]. Moreover, haploinsufficiency of SAM68 delayed mammary tumor onset and reduced metastasis [29]. Although the authors reported higher activation of SRC and FAK in the mammary gland of* Sam68* heterozygote females, indicating altered regulation of the SRC signal transduction pathway [29], whether or not this effect was related to the lower tumorigenicity of* Sam68* haploinsufficient cells was not investigated.

Additional phenotypes of the* Sam68−/−* mice revealed the important role played by this RBP in a number of physiological processes. Adult knockout females displayed defects in bone metabolism [28] and delayed development of sexual organs [29].* Sam68−/−* mice were protected against age-induced osteoporosis and were characterized by preserved bone density. This phenotype was linked to the preferential differentiation of knockout mesenchymal stem cells toward osteoblasts instead of adipocytes [28]. Furthermore, *Sam68−/−* females displayed a reduction in the number of developing ovarian follicles, alteration of estrous cycles, and impaired fertility [30]. Similarly, spermatogenesis and fertility were impaired in* Sam68−/−* males, due to the involvement of both nuclear RNA processing events [31] and translational regulation of a subset of mRNAs during spermiogenesis [32]. Although almost exclusively nuclear in the majority of normal cells, SAM68 localized in the cytoplasm of secondary spermatocytes and associated with polysomes, thus playing a role in translational regulation of target mRNAs [32, 33]. Notably, this function in male germ cells closely resembles that of its orthologue in* C. elegans* GLD-1.

Aberrant regulation of splicing events also contributes to the phenotypes of* Sam68−/−* mice. For instance, stimulation of* Sam68−/−* cerebellar neurons was dramatically attenuated due to the impaired regulation of* Nrxn-1* alternative splicing [34].* Nrxn-1* encodes a synaptic cell surface receptor that contributes to the assembly of functional presynaptic terminals, and a severe perturbation of* Nrxn-1* splice variants was observed in* Sam68−/−* brains [34]. Moreover,* Sam68−/−* mice exhibited a lean phenotype due to a dramatic reduction in adiposity. The decreased commitment to early adipocyte progenitors and defects in adipogenic differentiation were attributed to aberrant splicing of* mTOR* described in *Sam68−/−* mice [35].

Collectively, the defects documented in* Sam68* knockout mice reflect the multiple roles played by SAM68 in signal transduction and RNA processing and emphasize how aberrant regulation of SAM68 function(s) might contribute to oncogenic transformation [28, 29, 36]. Nevertheless, to what extent SAM68 RNA binding activity contributes to the mouse defects and to neoplastic transformation has not been unraveled yet, and, in this context, knock-in or transgenic mouse models displaying* Sam68* gene with mutations in the RNA binding domain would really help to answer this question.

## 3. SAM68 Signaling in Human Cancer

SAM68 acts as a scaffold protein in response to different signal transduction pathways [36, 41]. Through its proline-rich motifs, SAM68 interacts with the SH3 domains of different SRC kinases [1, 2], like BRK [42], FYN [18], and Itk/Tec/BTK [43], all involved in different aspects of cell transformation. Importantly, the interaction of SAM68 with the SRC SH3 domain enables SRC kinases to phosphorylate their substrates [9].

The interaction of SAM68 with FYN induces the assembly of a protein complex containing also PLC*γ*1 (phospholipase C gamma) [18], triggering its phosphorylation and activation [18, 44]. Interestingly, a truncated form of the tyrosine kinase receptor c-KIT, named tr-KIT, stimulates the formation of this complex [18]. Tr-KIT is aberrantly expressed in a subgroup of prostate cancer (PCa) patients and its expression correlates with enhanced activation of SRC and elevated expression and high tyrosine phosphorylation of SAM68 [45]. Moreover, SAM68 is frequently upregulated in PCa patients and promotes PCa cell proliferation and survival to chemotherapeutic agents [46], suggesting a role for this pathway in prostate cancer biology.

The breast tumor kinase BRK, a nonreceptor tyrosine kinase, is also responsible for the tyrosine phosphorylation of SAM68 in cancer cells, which has been associated with SAM68 increased nuclear localization and cell cycle promotion [47, 48]. Importantly, both SAM68 and BRK are upregulated in breast cancer cells and breast tumors [39, 48, 49]. In addition, in the transformed HT29 adenocarcinoma cell line, endogenous BRK colocalized in SAM68 nuclear bodies (SNBs), and BRK-mediated phosphorylation of SAM68 impaired its ability to bind RNA molecules [50]. Consistent with these results, nuclear BRK was also detected in differentiated androgen-responsive LNCaP human PCa cell line, while it was mainly cytoplasmic in the undifferentiated and more aggressive androgen-unresponsive PC3 prostate cancer cell line [50]. Thus, relocalization of the BRK kinase during PCa development and progression may indicate disruption of a signaling pathway important for maintaining the normal phenotype of prostate epithelial cells.

Proteomic analyses revealed that SAM68 is able to form two (large and small) protein complexes, interacting with several RBPs and with regulators of cytoskeletal organization and signal transduction pathways [51, 52]. In accordance with this,* SAM68*-deficient fibroblasts displayed defects in cell migration [53] and an increase in SRC kinase activity [53]. These observations suggest that SAM68 is required for a negative feedback inhibition of SRC and that deregulated SRC activity could be responsible for the defects in actin cytoskeleton and cell migration observed in* SAM68*-deficient fibroblasts. Interestingly, epidermal growth factor (EGF) treatment induced a change in the size of the SAM68-containing complexes, from the large to the smaller one, the latter containing splicing activity [51]. Since EGF receptor (EGFR) stimulation triggers signaling cascades controlling cellular proliferation, migration, differentiation, and survival, and EGFR overexpression has been associated with poor prognosis in several types of epithelial cancers, such as lung, head and neck, colorectal, and breast cancer [54], EGFR-SAM68 signaling could be targeted to attenuate the oncogenic features of cancer cells.

In addition to PCa [46, 52], aberrant expression of SAM68 was detected in several other tumors. In particular, SAM68 was shown to be upregulated in colorectal cancer [55] and in patients with non-small cell lung cancer [56]. Moreover, in patients with renal cell carcinoma high SAM68 expression was inversely associated with overall survival while SAM68 cytoplasmic localization significantly correlated with pathologic grade and outcome of this tumor [57]. Furthermore, in breast cancer patients expression and cytoplasmic localization of SAM68 significantly correlated with clinical characteristics of patients, including clinical stage, tumour-nodule-metastasis classification, histological grade, and ER expression [39]. In line with an oncogenic role played by SAM68 in this tumor type, silencing of SAM68 inhibited proliferation and tumourigenicity of breast cancer cells [39]. Finally, SAM68 was shown to be significantly upregulated in cervical cancer at both mRNA and protein levels [58]. SAM68 upregulation and its cytoplasmic localization were significantly associated with risk factors and correlated with lymph node metastasis and poor prognosis in patients with early-stage cervical cancer [58]. Consistently, downregulation of SAM68 in cervical cancer cells inhibited cellular motility and invasion by the inhibition of the AKT/GSK-3*β*/Snail pathway [58].

Collectively, these reports strongly suggest that high SAM68 expression and its cytoplasmic localization are associated with poor overall survival in different types of tumors. Moreover, the deregulation of SRC and AKT pathways could be involved in the oncogenic function of SAM68 in the cytoplasm.

## 4. SAM68 and Transcriptional Regulation in Human Cancer

**Figure 2:**
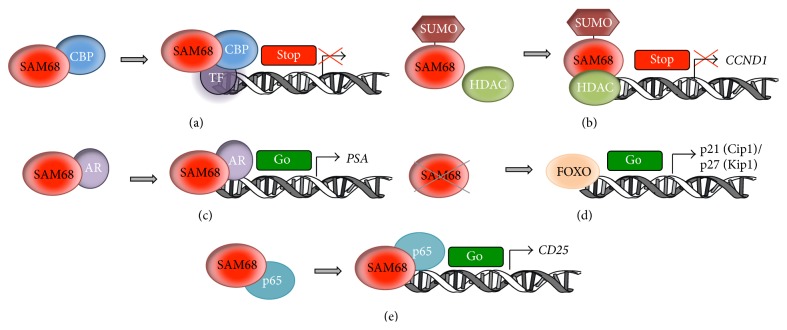
Transcriptional regulation by SAM68. (a) SAM68 forms a complex with CBP and transcriptional repressor factors (TF), thus negatively regulating CBP targets transcription [37]. (b) The PIAS1 complex SUMOylates SAM68, which interacts with a histone deacetylase (HDAC) and represses* CCDN1* transcription [23]. (c) SAM68 directly interacts with the androgen receptor (AR) and binds to androgen-responsive elements (AREs) leading to AR targets activation (i.e.,* PSA* gene) [38]. (d) SAM68 depletion in breast cancer cells leads to activation of FOXO factors thus inhibiting cell proliferation and tumourigenicity through the upregulation of cyclin-dependent kinase inhibitors p21 (Cip1) and p27 (Kip1) [39]. (e) SAM68 binds the* CD25* promoter and facilitates p65 recruitment, thus contributing to NF-*κ*B regulation of gene expression [40].

The first evidence of the involvement of SAM68 in transcriptional regulation came out in 2002 when Hong and colleagues documented the repressive effect of SAM68 on different mammalian and viral promoter constructs [37]. Direct recruitment of SAM68 to a promoter region resulted in strong transcriptional repression and mutation of the SAM68 RNA binding domain had no influence on this effect, thus suggesting that SAM68 transcriptional activity occurs in a RNA-independent fashion [37]. Mechanistically, the authors described the functional association of SAM68 with the acetyl-transferase CBP, which caused modulation of CBP transcriptional activity (Figure 2(a)) [37].

Other reports confirmed the role of SAM68 as a transcriptional repressor. SAM68 was shown to interact with hnRNP K, leading to inhibition of the* trans*-activating effects of hnRNP K on c-myc target genes [59]. Moreover, overexpression of SAM68 in mouse fibroblasts inhibited accumulation of* Cyclin D1* and* E* transcripts [60], whereas SAM68 SUMOylation by PIAS1 further enhanced repression of Cyclin D1 expression (Figure 2(b)) [23].

In PCa cells, SAM68 was proposed to function as a transcriptional coregulator and to promote the transcriptional activity of the androgen receptor (Figure 2(c)) [38]. Furthermore, in hematopoietic stem cells SAM68 was shown to form an oncogenic transcriptional complex with mixed lineage leukaemia (MLL) and PRMT1 [61]. Chimeric fusion of MLL with PRMT1 or SAM68 enhanced self-renewal of primary hematopoietic cells; conversely, specific knockdown of PRMT1 or SAM68 suppressed MLL-mediated oncogenic transformation [61]. Similarly, SAM68 depletion in breast cancer cells impaired cell proliferation and their tumorigenic features through the upregulation of cyclin-dependent kinase inhibitors p21 (Cip1) and p27 (Kip1). Thus, in this context SAM68 depletion might lead to suppression of AKT phosphorylation and subsequent activation of FOXO factors, which in turn promote the upregulation of p21 (Cip1) and p27 (Kip1) (Figure 2(d)) [39].

In normal and transformed human T cells SAM68 was shown to bind the* CD25* promoter and facilitate p65 recruitment, thus suggesting a novel role for SAM68 in NF-*κ*B regulation of gene expression in human T cell signaling (Figure 2(e)) [40]. In this context,* CD25* expression and aberrant NF-*κ*B signaling led to increased proliferation, expression of antiapoptotic proteins, and drug resistance, while* SAM68* knockdown markedly impaired CD25 upregulation. Remarkably, elevated expression of CD25 has been detected in a large variety of hematopoietic malignancies and solid tumors [62]; thus the p65-SAM68 association might be strategically used to target CD25 expression in those particular tumors that depend on CD25 for survival [40].

Transcription and RNA processing machineries are tightly coupled. Temporal coupling not only provides efficient gene expression to accomplish rapid growth and proliferation, but also allows rapid response to diverse signaling events [63]. Many splicing regulators are recruited to nascent pre-mRNAs by their interaction with the phosphorylated carboxyl-terminal domain (CTD) of RNAPII thus affecting splicing decisions [64]. Interestingly, SAM68 was shown to interact directly with RNA polymerase II (RNAPII) in meiotic spermatocytes [31] and with the RNAPII associated Brahma (Brm) subunit of the SWI/SNF chromatin-remodeling complex [65]. These observations strongly suggest the involvement of SAM68 in cotranscriptional splicing. Thus, on one hand, SAM68 binding to transcription factors and to the RNAPII itself can affect transcriptional regulation of gene expression; on the other hand, through the cooperation with chromatin remodelers, SAM68 can impact cotranscriptional splicing events. In this regard, interaction of the protooncogenic transcription factor FBI-1 with SAM68 in PCa cells was shown to inhibit SAM68 recruitment on the* BCL-X* pre-mRNA, thus affecting apoptosis [66]. By contrast, binding of SAM68 to the transcriptional coactivator SND1 was required for the efficient association of SAM68 with RNAPII and for the recruitment of SAM68 on the* CD44* pre-mRNA [67]. Remarkably,* CD44* alternative splicing isoforms are associated with tumor progression and metastasis [68]. Thus, the SND1/SAM68 complex might be an important determinant of PCa progression and the concomitant upregulation of these proteins might provide an advantage for cancer cells to invade other tissues, consequently favoring the spreading of metastatic cells [67].

Hence, depending on the cellular partner, SAM68 displays different effects on target genes, modulating in this way different or even antagonistic functions within the cell.

In summary, growing evidence documents the involvement of SAM68 in the transcriptional regulation of gene expression of cancer related genes, both by direct binding to the chromatin and by recruitment of specific transcription factors, which in turn affect its splicing activity.

## 5. SAM68-Regulated Alternative Splicing Events in Cancer

SAM68 preferentially binds A/U-rich sequences in RNA [16]. SELEX experiments identified the UAAA consensus motif bound with Kd ~12–60 nM. Importantly, a single A to C mutation within this motif abolished SAM68 binding [69], indicating that this motif is involved in high affinity direct binding or in a specific RNA structure. Indeed, SAM68 was then shown to bind cellular RNAs enriched in such U/A-rich sequences [70] and to directly modulate alternative splicing events in target genes [71]. Interestingly, the UAAA motif matches with the last four bases of the mammalian polyadenylation signal AAUAAA, thus opening the hypothesis of SAM68 involvement in RNA stability.

**Figure 3:**
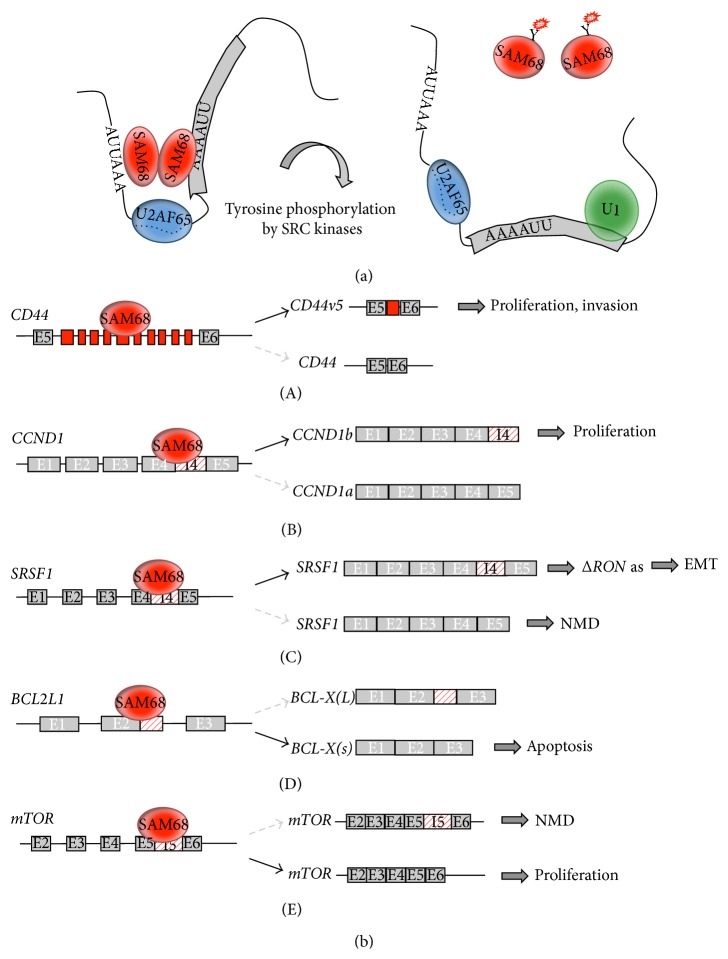
Model of SAM68 interaction with pre-mRNAs and splicing regulation. (a) SAM68 recognition of A/U-rich bipartite sequence in the pre-mRNA homodimerization allows simultaneous binding to the pre-mRNA and to U2AF65 [69–73]. Tyrosine phosphorylation of SAM68 reduces the RNA binding affinity and thus releases SAM68 from the pre-mRNA. (b) Model of alternative splicing events regulated by SAM68 in cancer cells. (A) SAM68 promotes inclusion of* CD44* variable exon* v5*. Inclusion of variable exons in the* CD44* pre-mRNA is specific to cancer cells and correlates with cancer progression and invasiveness [71]. (B) SAM68 promotes splicing events that regulate cell proliferation. Binding of SAM68 to* CCND1* intron 4 interferes with the correct recruitment of U1 snRNP at the exon 4 5′ splice sites, thus enhancing retention of intron 4 and generating the* Cyclin D1b* isoform. In prostate cancer, the expression of Cyclin D1b interrupts a negative feedback in the regulation of androgen receptor (AR) transcriptional activity, thereby promoting cell proliferation [74]. (C) As for* CCND1*, SAM68 promotes retention of* SRSF1* intron 4, thus stabilizing* SRSF1* pre-mRNA and inhibiting its degradation by nonsense-mediated decay (NMD) [75]. Accumulation of SRSF1 in turn favors the splicing of* ΔRON*, an oncogenic variant of* RON* that triggers epithelial-mesenchymal transition (EMT). (D) SAM68 regulates the alternative splicing of* BCL2L1* leading to the short (*BCL-X(s)*) proapoptotic isoform [19]. This activity can be reverted by tyrosine phosphorylation of SAM68 from SRC family kinases, thereby switching the role of SAM68 from being proapoptotic to being antiapoptotic and allowing cells to differentially react to external cues. (E) SAM68 regulates* mTOR* alternative splicing thus leading to the correct mRNA isoform and avoiding retention of intron 5 that generates a premature termination codon and the consequent reduction of mTOR protein levels [35]. Notably, mTOR is a critical effector in cell-signalling pathways commonly deregulated in human cancers and overexpression of the components involved in the PI3K/AKT/mTOR pathway has been shown to induce malignant transformation.

During tumor progression, a variety of oncogenic signaling pathways induce modifications of the downstream effectors of key biological functions [76]. Notably, SAM68 was the first identified “hub factor” able to translate extracellular stimuli to pre-mRNA processing of specific target genes in the nucleus [71]. As mentioned above, several posttranslational modifications regulate the function and/or localization of SAM68. In particular, serine-threonine and tyrosine phosphorylation of SAM68, which often occurs in cancer cells, are important for SAM68 homodimerization and RNA affinity (Figure 3(a)) [2, 72, 73].

The* CD44* gene represents an interesting example of SAM68-mediated coupling between signal transduction cascades and alternative splicing.* CD44* pre-mRNA is affected by complex alternative splicing events occurring in 10 adjacent exons (v1–v10) to produce multifunctional transmembrane glycoprotein isoforms implicated in cell-cell and cell-matrix adhesion, migration, and invasion [77] and with crucial roles in cancer progression and metastasis [78]. By binding to A/U-rich enhancer element located within exon* v5*, SAM68 promotes the production of the oncogenic* CD44v5* variant (Figure 3(b), (A)) [71], which is upregulated in several cancers [78, 79] and bears prognostic value in gastric and renal carcinoma [80–82].

Several molecular mechanisms (not mutually exclusive) have been proposed to explain the ability of SAM68 to stimulate* CD44* exon* v5* inclusion: (i) SAM68 competes or displaces the antagonistic splicing repressor hnRNP A1 that binds a specific splicing silencer element located within exon* v5* [83]; (ii) SAM68 affects the dynamic recruitment of spliceosomal components, including U2AF65, an auxiliary factor involved in the recognition of the 3′ splice site during the splicing reaction [84]; upon SAM68 phosphorylation this interaction is disrupted and U2AF65 dissociates from pre-mRNA allowing the subsequent spliceosome remodeling and exon* v5* inclusion [85]; (iii) SAM68 interacts with the splicing coactivator SRm160 and they functionally cooperate to simulate* CD44* exon* v5* inclusion [86].

Aberrant regulation of alternative splicing is emerging as a key step in oncogenesis [87]. Recent data demonstrated that genotoxic stress widely modulates alternative splicing events in cancer cells [88, 89]. This regulation is exerted in part through reduced transcription elongation rates as a consequence of RNA polymerase II (RNAPII) phosphorylation [90] and in part through direct involvement of specific RBPs in the repair process or by specific regulation of DNA damage response gene expression [91], also accomplished by RBP relocalization [92].* CD44* exon* v5* splicing is also influenced by genotoxic stress induced by chemotherapeutic drugs, such as the topoisomerase II inhibitor mitoxantrone (MTX) [93]. Specifically, MTX causes relocalization of SAM68 from nucleoplasm to transcriptionally active nuclear granules and this correlates with changes in alternative splicing of* CD44* exon* v5*. This effect is independent of signal transduction pathways activated by DNA damage [93]. Nevertheless, it appears to be functionally relevant for the cells, as SAM68 was found overexpressed in prostate carcinoma where it promotes resistance and survival to chemotherapeutic treatments [46].

In addition to* CD44*, changes in alternative splicing of other transcripts, including* Caspase 2* (*CASP2*) [94],* BCL-2* [90], the p53 negative modulators* MDM2* and* MDM4* [95], and* Cyclin D1* (*CCND1*), have been observed in cancer cells after treatment with chemotherapy drugs [96, 97]. Notably,* CCND1* pre-mRNA was also identified as a novel alternative splicing target of SAM68 [74].* CCND1* is a protooncogene that is frequently deregulated in several human cancers through different mechanisms, such as chromosomal translocations, amplification of the* CCND1* locus, and intragenic mutations [97–99]. Alternative splicing also plays an important role in aberrant Cyclin D1 expression. The* CCND1* gene encodes two alternatively spliced transcripts: the canonical* Cyclin D1a* and the alternative* Cyclin D1b*, which results from the retention of intron 4 and premature termination of the transcript [100]. These isoforms display different biological properties and cellular localization [96]. In particular, Cyclin D1b is exclusively nuclear and displays stronger oncogenic potential than Cyclin D1a [74, 100, 101] and its upregulation correlates with poor prognosis in several tumor types [96]. At the molecular level, SAM68 was observed to bind to the proximal region of intron 4 and to interfere with the recruitment of the U1 snRNP, in this way promoting intron 4 retention (Figure 3(b), (B)) [74]. Signal transduction pathways affecting SAM68 phosphorylation status, such as those conveyed by ERK1/2 and SRC kinases, regulate alternative splicing of* CCND1* pre-mRNA by modulating SAM68 affinity for this target [74]. Notably, SAM68 expression positively correlates with levels of Cyclin D1b, but not D1a, in human PCa cells [97], suggesting that increased levels of SAM68 in human PCa contribute to tumorigenesis by elevating the expression of Cyclin D1b in this tumor type.

Recent studies have demonstrated an important contribution of alternative splicing regulation in the cascade of events characterizing the morphological conversion of tumor cells during epithelial-to-mesenchymal transition (EMT) [102], one of the major routes through which cancer cells acquire migratory and invasive potentials [103, 104]. SAM68 phosphorylation by ERK1/2 plays an important role during neoplastic progression of epithelial cells through activation of EMT. This is illustrated by the ability of SAM68 to repress alternative splicing-activated nonsense-mediated mRNA decay (AS-NMD) [105] of a splicing factor of the serine arginine (SR) family, SRSF1 [75]. AS-NMD of* SRSF1* pre-mRNA, which involves a cryptic intron in the 3′ UTR region of the gene, decreases* SRSF1* mRNA stability and protein levels (Figure 3(b), (C)) and, notably, this event is altered in colon cancer [75]. In mesenchymal cells, phosphorylation of SAM68 is controlled by soluble factors expressed by epithelial cells that act through the activation of ERK1/2 kinase [75]. SRSF1, an oncogenic splicing factor upregulated in many human cancers [106], severely impacts on cell physiology. For instance, its overexpression stimulates skipping of exon 11 of the* RON* protooncogene increasing the production of the constitutively active* ΔRON *isoform, which in turn promotes the acquisition of an invasive cellular phenotype [107]. Interestingly, inhibition of ERK activity by small molecules or by using conditioned medium from epithelial cells reverts SAM68 phosphorylation, decreases* SRSF1* mRNA and protein levels, promotes inclusion of* RON* exon 11, and induces the reversal program named mesenchymal-to-epithelial transition (MET) [75]. MET occurs at the final metastatic sites where redifferentiation of mesenchymal cells to an epithelial state is required for the colonization of distant organs [103, 104].

A paradigmatic example of the central role of SAM68 in apoptosis is represented by the regulation of* BCL-X* (*BCL2L1*), a member of the* BCL-2* gene family.* BCL-X* pre-mRNA is alternatively spliced to generate two isoforms with opposite functions in promoting apoptosis. Selection of the proximal 5′ splice site (5′ SS) in exon 2 causes the production of the antiapoptotic long* BCL-X(L)* variant, while the proapoptotic short* BCL-X(s)* variant is produced by the use of the distal alternative 5′ SS [108]. In several cancer types, the* BCL-X(L)* isoform is upregulated thus increasing resistance to chemotherapeutic agents [109, 110]. Targeting this mechanism and switching the splicing of* BCL-X* gene toward the production of the proapoptotic variant thereby offer the opportunity to revert cancer cells resistance to chemotherapeutic drugs and to promote tumor cell death [111, 112]. Due to its relevance in cancer,* BCL-X* alternative splicing has been extensively investigated in the past years and several RBPs were shown to regulate this specific splicing event [19, 113–119]. Among these, SAM68 exerts a proapoptotic function, leading to production of* BCL-X(s)* variant [19]. In particular, SAM68-mediated splicing regulation of* BCL-X* depends on its specific binding to* BCL-X* pre-mRNA and on its ability to interact with the splicing repressor hnRNP A1, thus antagonizing SRSF1, a positive regulator of* BCL-X(L)* splicing (Figure 3(b), (D)) [19, 110]. However, in PCa cells, high levels of SAM68 do not correlate with high levels of* BCL-X(s)* [38, 46, 110]. This apparently contradictory observation can be explained by the fact that tyrosine phosphorylation of SAM68 by the SRC-related kinase FYN counteracts its splicing activity, promoting the antiapoptotic* BCL-X(L)* isoform [19, 120]. In tumors, SRC activity is often increased [121] and it correlates with SAM68 phosphorylation in different cancer types, including prostate cancer [45, 47, 122]. Recently, an additional layer of complexity to the regulation of SAM68-mediated* BCL-X* splicing in cancer has been revealed. This mechanism involves the direct interaction of the transcriptional factor FBI-1 with SAM68, reducing its binding to* BCL-X* pre-mRNA and therefore promoting the production of the antiapoptotic* BCL-X(L)* variant and cell survival [66]. Fascinatingly, FBI-1 function in* BCL-X* splicing regulation is dependent on the activity of histone deacetylases [66], suggesting an important link between this alternative splicing event and dynamic organization of chromatin structure.

The biological consequences and the possible contribution to tumor progression associated with the aberrant splicing in other relevant SAM68-regulated genes have also been recently described. For example, SAM68 is able to promote the production of the oncoprotein E6 of the human papilloma virus (HPV) type 16 [123], which is a known etiological agent for human cervical cancer [124].* E6* alternative splicing is controlled by EGF through activation of ERK1/2-kinase that promotes SAM68 phosphorylation, suggesting a possible implication of SAM68 in HPV* E6* splicing during differentiation and the viral life cycle processes of cervical cancer.

More recently, SAM68 has been linked to regulation of alternative splicing of the mammalian target of rapamycin (mTOR) [35], which regulates cell size and cell proliferation in response to nutrients and various growth factors [125, 126]. SAM68-depleted cells display intron 5 retention in the* mTOR* mRNA, which generates a premature termination codon and the consequent reduction of mTOR protein levels (Figure 3(b), (E)) [35]. Notably, mTOR is a critical effector in cell-signaling pathways commonly deregulated in human cancers and overexpression of the components involved in the PI3K/AKT/mTOR pathway has been shown to induce malignant transformation [127]. Interestingly, loss of SAM68 reduces breast and PCa incidence [29, 46], suggesting that in cancer cells SAM68 activation may also regulate the expression of PI3K downstream kinases, such as mTOR.

Collectively, these findings indicate that an evaluation of SAM68-associated splicing signatures in diverse sets of tumors can be of medical relevance.

## 6. SAM68 and Noncoding RNAs

Recent reports have revealed the involvement of SAM68 in noncoding RNAs (ncRNAs) metabolism. ncRNAs are classified into small (18–200 nt) and long ncRNAs (lncRNAs; 200 nt to >100 kb) [128, 129] and play a role in a wide variety of biological processes, including almost all levels of gene expression regulation, from epigenetic to transcriptional and posttranscriptional control [130]. Coimmunoprecipitation studies documented the interaction between SAM68 and key proteins involved in microRNA (miRNA) biogenesis [131]. miRNA genes are transcribed by either RNA polymerase II or RNA polymerase III into long primary miRNA transcripts (pri-miRNAs) [132]. The cleavage of the pri-miRNAs into stem-loop precursors of ~70 nucleotides (pre-miRNAs) is mediated by DROSHA [133], whereas the cytoplasmic processing of pre-miRNAs into mature miRNAs is mediated by DICER [134]. Coimmunoprecipitation experiments performed in male germ cells indicated that SAM68 interacts with both DICER and DROSHA and that the knockout of* Sam68* leads to changes in expression of specific miRNAs in germ cells [131]. Remarkably, a similar functional interaction with components of the miRNA machinery was shown for Quaking (QKI), another member of the STAR family. In the U343 glioblastoma cell line and in primary rat oligodendrocytes QKI interacts with AGO2, a component of the RISC complex involved in miRNA-dependent translational repression, within stress granules [135]. Collectively, these findings suggest a general role for STAR proteins in the regulation of miRNAs.

Interaction between SAM68 and noncoding RNAs might also affect the splicing activity of this RBP. Recently, a long noncoding RNA (named* INXS*) has been described as a novel mediator of SAM68-dependent regulation of* BCL-X* splicing.* INXS* is transcribed from the antisense genomic strand of* BCL-X* gene and is downregulated in various tumor cell lines and in kidney tumor tissues, whereas its expression is induced by treatments that trigger apoptosis [136].* INXS* interacts with SAM68 and favors its splicing activity, thus increasing the levels of* BCL-X(s)* isoform and enhancing apoptosis [136]. Notably, in favor of a possible role of* INXS* in anticancer therapy,* INXS* overexpression in a mouse xenograft model was sufficient to induce tumor regression and increase* BCL-X(s)* isoform [136].

Thus, the complex regulatory network of proteins and ncRNAs orchestrated by SAM68 greatly contributes to the cellular signature in higher eukaryotes and plays a pivotal role in the regulation of gene expression in normal conditions and in oncogenic transformation.

## 7. Concluding Remarks

**Figure 4:**
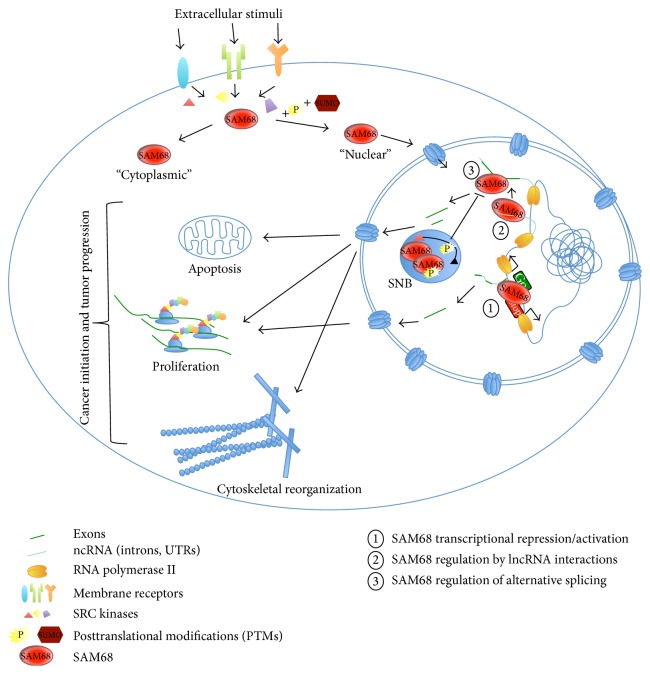
Role(s) of SAM68 in transcriptional and posttranscriptional regulation of gene expression in cancer cells. SAM68 and its regulatory networks contribute to important process involved in cancer initiation and progression, such as apoptosis, proliferation, and cytoskeletal reorganization, through different mechanisms. After posttranslational modifications (PTMs) induced by extracellular stimuli and mediated by SRC family kinases, SAM68 is committed to the nucleus where it is able to (1) promote or repress transcription of different targets (see Figure 2 for more details) and (2-3) regulate alternative splicing events through several molecular mechanisms, some of them mediated by lncRNAs (see Figure 3 for more details). In the nucleus, SAM68 can localize in specific bodies (SNB) and associate with other proteins (i.e., BRK kinase) that modify its phosphorylation status, thus affecting its RNA binding activity.

Misregulation of cancer-associated alternative splicing events is often correlated with unbalanced expression of splicing factors. SAM68 is a clear example of this concept, as it is upregulated in different types of tumors and it directly affects cancer initiation and progression. Transcriptional and posttranscriptional regulation of gene expression mastered by SAM68 chiefly contributes to changes in gene expression occurring in cancer cells. Moreover, SAM68 orchestrates transcript fate and function (Figure 4). Thus, depicting SAM68 signatures in normal and cancer cells would greatly help in understanding how SAM68 and its regulatory networks contribute to key features of tumor initiation and progression. Although the functional significance of SAM68-regulated alternative splicing events in human cancer has been clearly established, future studies unraveling the positional effect of SAM68 binding to pre-mRNAs would be instrumental for the development of new therapeutic approaches to target SAM68 activities in cancer.
